# LANet: Stereo matching network based on linear-attention mechanism for depth estimation optimization in 3D reconstruction of inter-forest scene

**DOI:** 10.3389/fpls.2022.978564

**Published:** 2022-09-02

**Authors:** Lina Liu, Yaqiu Liu, Yunlei Lv, Jian Xing

**Affiliations:** College of Information and Computer Engineering, Northeast Forestry University, Harbin, China

**Keywords:** forestry 3D reconstruction, depth estimation, stereo match, linear-attention, self-attention, stacked hourglasses

## Abstract

The 3D reconstruction of forests provides a strong basis for scientific regulation of tree growth and fine survey of forest resources. Depth estimation is the key to the 3D reconstruction of inter-forest scene, which directly determines the effect of digital stereo reproduction. In order to solve the problem that the existing stereo matching methods lack the ability to use environmental information to find the consistency of ill-posed regions, resulting in poor matching effect in regions with weak texture, occlusion and other inconspicuous features, LANet, a stereo matching network based on Linear-Attention mechanism is proposed, which improves the stereo matching accuracy by effectively utilizing the global and local information of the environment, thereby optimizing the depth estimation effect. An AM attention module including a spatial attention module (SAM) and a channel attention module (CAM) is designed to model the semantic relevance of inter-forest scenes from the spatial and channel dimensions. The linear-attention mechanism proposed in SAM reduces the overall complexity of Self-Attention from O(*n*^2^) to O(n), and selectively aggregates the features of each position by weighted summation of all positions, so as to learn rich contextual relations to capture long-range dependencies. The Self-Attention mechanism used in CAM selectively emphasizes interdependent channel maps by learning the associated features between different channels. A 3D CNN module is optimized to adjust the matching cost volume by combining multiple stacked hourglass networks with intermediate supervision, which further improves the speed of the model while reducing the cost of inferential calculation. The proposed LANet is tested on the SceneFlow dataset with EPE of 0.82 and three-pixel-error of 2.31%, and tested on the Forest dataset with EPE of 0.68 and D1-all of 2.15% both of which outperform some state-of-the-art methods, and the comprehensive performance is very competitive. LANet can obtain high-precision disparity values of the inter-forest scene, which can be converted to obtain depth information, thus providing key data for high-quality 3D reconstruction of the forest.

## Introduction

Three-dimensional scene reconstruction is an important research direction in the field of computer vision, which is widely used in popular fields such as object detection and recognition, automatic driving and robot navigation. The 3D reconstruction of an inter-forest scene uses binocular cameras and UAVs to take low-altitude photography from different perspectives to realistically reproduce the 3D structure of forest objects, effectively solving the problems of limited vision, overlapping and obscuring trees, artificial inaccessibility, harsh conditions, dangerous environment and other survey difficulties. The visual forest scene clearly and comprehensively shows the structural information of trees, such as the trunk, main branches, side branches, tree height, crown height, and crown width. Professionals analyze the structural characteristics, spatial isolation degree, size differentiation degree and horizontal distribution pattern of trees through the survey of the tree structure and its surrounding environment, which provides a strong basis for scientific regulation of tree growth, optimization of forest structure and fine survey of forestry resources such as stand volume and stand density, and plays an important role in evaluating the economic, ecological and social value of forests.

Scene depth estimation is a key step in the 3D reconstruction of the forest, which directly determines the effect of 3D reconstruction. Binocular stereo matching imitates human binocular perception by finding the corresponding points between the left and right image planes and using the geometric relationship of the corresponding points to obtain the disparity value d. For the pixel point (x, y) in the left image, the coordinates of its corresponding point in the right image are (x-d, y), and the disparity value can be converted into the depth information of the scene by F^*^L/d, where F represents the focal length of the camera and L is the distance between the two camera centers. The binocular stereo matching method has high matching accuracy and speed, and the binocular camera has the advantages of easy portability, flexible operation and low cost. Its non-contact and non-radiation characteristics can achieve 3D environment perception in the forest without causing any damage to the environment, and maximize the protection of the forest's ecological environment. James Garforth (Garforth and Webb, 2019), University of Edinburgh, UK, pointed out that the use of vision sensors for 3D reconstruction of forest scenes, and based on this, forest resource information collection and intelligent forestry robots for navigation, positioning and operational target identification are the most promising methods.

Gatziolis et al. (2015) developed a system for accurately acquiring 3D models of trees by using a small UAV with a lightweight, inexpensive camera that moves slowly along a predetermined trace to acquire images, and by using computer vision methods to process the images to obtain detailed 3D structures of the trees. Ni et al. (2016) used binocular stereo vision to recover 3D information on tree crowns. Using multi-view acquisition of the target images, combining the SfM method to recover the camera calibration matrix of each image, to achieve a sparse projection reconstruction of the target, and using a spherical pivot algorithm for surface modeling, to achieve a dense reconstruction of the tree crown. Finally, the reconstruction is converted to a metric by obtaining ground truth points in camera calibration. Zhang (2003) used an improved SURF algorithm to find the feature points in the two images and designed a matching strategy suitable for tree trunk edges. Zhang then performed a 3D reconstruction of the tree and developed a system for close-up photography and stereo measurement of trees (FVision). Han (2003) used the camera of a mobile phone as a device for tree image acquisition, and reconstructed the 3D structure of trees, calculating tree-measurement factors such as tree height, tree diameter, and wood volume. Malekabadi et al. (2019) used a stereo vision system to obtain tree disparity maps to analyse the potential of geometric properties. Xu (2015) built a parallel binocular vision platform, marked four rectangular red information points on trees, extracted the coordinates of the information points using the merging algorithm based on membership degree and 2D maximum entropy theory, and realized the inverse study of tree growth such as tree height and wood volume based on the incremental changes of each information point within a year. Zhang et al. (2022) built a binocular vision-based shape reconstruction and measurement system for front-end vision information of spherical hedges, improved semi-global block matching (SGBM) algorithm to get a disparity map of spherical hedges, according to the disparity map and parallel structure of the binocular vision system, the 3D point cloud of the target is obtained.

At present, there are still relatively few studies at home and abroad on the use of binocular vision methods for rapid 3D reconstruction of forest scenes. However, with the wide application of deep learning in the fields of target recognition, semantic segmentation and natural language processing, the application of deep learning to stereo matching has explored richer feature representation and aggregation algorithms, which has greatly improved the performance of stereo matching compared to traditional methods.

The application of CNNs to stereo matching was first proposed by Zbontar and LeCun (2016), who designed a deep twin network Siamese to compute the matching cost, using traditional crossover-based cost aggregation and semi-global matching methods to process the matching cost to obtain the disparity map. Shaked and Lior (2017) proposed to replace two steps in the traditional algorithmic process with two deep neural networks: a highway network to calculate the matching cost and a global disparity network to obtain the initial disparity map and the confidence degree of the predicted result, which would facilitate better detection of anomalies in the subsequent disparity correction step. With the development of fully convolutional neural networks (FCNs) (Long et al., 2015), it has been used in pixel-level labeling task to learn disparity map from end-to-end and achieved good results. Mayer et al. (2016) proposed the first end-to-end network, DispNet, which outputs a predicted disparity map by feeding a pair of binocular image pairs through an hourglass-type “encoder-decoder” architecture. Pang et al. (2017) extended the basis of DispNet by proposing a two-stage composition of the stacked network (CRL) cascaded residual learning: the first stage is used to regress the initial disparity, and the second stage corrects the initial disparity generated from the first stage to form multi-scale residuals, and finally the outputs of the two stages are summed to form the final disparity map. Recent research study has mostly used end-to-end disparity maps regression based on a series of feature volume and 3D aggregation networks for better contextual aggregation. GC-Net (Kendall et al., 2017) innovatively proposed the form of cost volume based on the end-to-end network architecture of DispNet, where the cost volume of 4D is obtained by concatenating the left feature with their corresponding right feature from across each disparity level. The 3D convolution network is the first used to learn the global context information from neighbor pixels and disparities to predict the disparity probability. PSMNet (Chang and Chen, 2018) uses a pyramidal pooling layer, SPP, and a 3D CNN to replace the feature extraction and cost matching modules in GC-Net, SPP can make full use of global environmental information by aggregating environmental information at different scales and locations to build a matching cost volume. The 3D CNN adjusts the matching cost volume by combining multiple stacked hourglass networks with intermediate supervision, which enables the PSMNet to make fuller use of contextual information compared to the GC-Net approach.

However, the above methods all have their own shortcomings. The stereo matching algorithm of CNN is limited by the perceptual field of the convolutional network and still has a large number of incorrect matching results in such ill-posed regions as weak textures and reflection. DispNet does not combine different scales and different location information to construct matching costs and lacks contextual information features. GC-Net uses a 4D cost volume to represent the correspondence between left and right images, and uses 3D CNNs to learn global contextual information in both the spatial dimension and the disparity dimension, but does not consider the correlation between contexts. PSMNet uses average pooling to compress features to four scales, up-sampling by bilinear interpolation, and expanding the receptive field by dilated convolution, but increasing the receptive field size is not equivalent to capturing the correlation between contexts, and it ignores the contribution of distant pixels to the current region, thus lacking the interaction between local information and the long-range dependence of global network information.

The attention mechanism (Zhang et al., 2019) is able to capture rich contextual relevance by learning contextual information and adaptively integrating local and global information, which compensates well for the limitations of convolutional operations. DANet (Fu et al., 2019) uses a Self-Attention mechanism to integrate contextual information to achieve good results for the segmentation task of scenes.

Combining the experience of scene segmentation with the idea of making full use of local and global environmental information on the whole image, we apply it to the depth estimation of complex forest scenes and propose a stereo matching network LANet based on the Linear-Attention mechanism. Our main contributions can be summarized as follows.

1. An innovative forestry application is proposed for fine surveying in the forest: LANet, an end-to-end stereo matching network is proposed to obtain disparity maps for forest scenes. An AM attention module including SAM and CAM is designed to obtain a rich representation of pixel-level features of forest scenes.
2. A Linear-Attention mechanism is proposed in SAM, which captures long-range dependencies by learning rich contextual relationships while reducing the overall complexity of Self-Attention from O(*n*^2^) to O(n) in both time and space. Self-Attention is used in CAM to selectively emphasize interdependent channel maps by learning the correlation between different channel features, thereby improving feature discrimination.
3. Optimizing stacked 3D hourglasses reduces the computational cost and improves inference speed by combining multiple stacked hourglass networks with intermediate supervision to adjust matching cost volumes, and using 1 × 1 × 1 3D convolutions in shortcut connections within each hourglass module, removing shortcut connections between different output modules of the hourglass.

## Methodology

**Figure 1:**
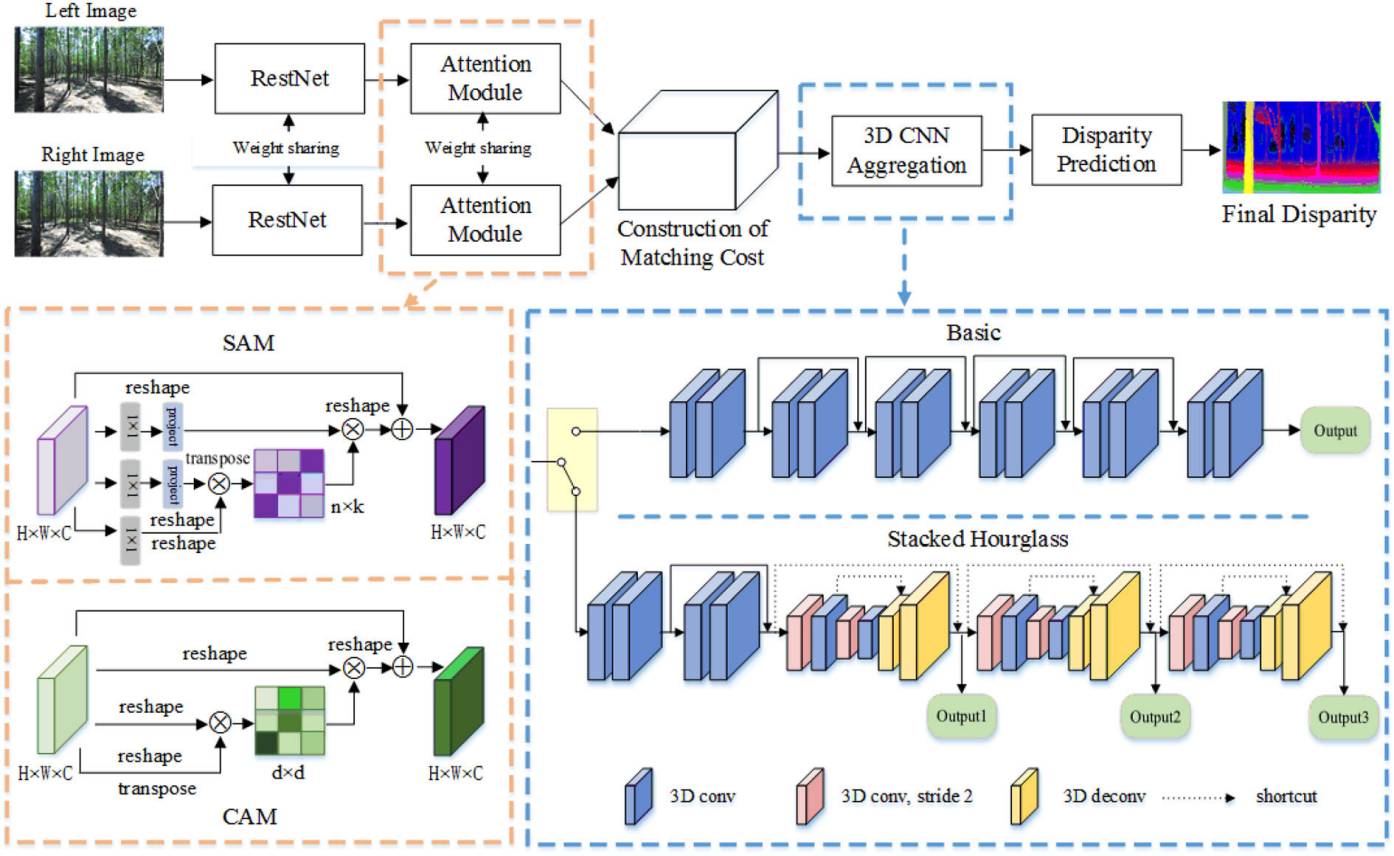
Architecture overview of proposed LANet.

The LANet that we propose for depth estimation optimization in the 3D reconstruction of the inter-forest scene consists of five parts: ResNet, Attention Module (AM), Construction of Matching cost, 3D CNN Aggregation, and Disparity Prediction, as illustrated in Figure 1. Details of this model are provided as follows.

### Details of the network structure

Table 1 lists the layers of each module and the corresponding parameter settings. H, W denotes the height and width of the image, C denotes the number of channels, D denotes the maximum disparity, and S1 and S2 denote convolution stride. Each 3D convolution by default contains batch normalized BN and linear activation ReLU,^*^ indicate that ReLU is not included. ^**^ indicate that ReLU and BN are not included, only convolution.

**Table 1:** Layers and parameter settings of the proposed LANet.

**Module**	**Layer**	**Setting**	**Output size**
ResNet	conv0_1	3 × 3,32,S2	1/2H × 1/2W × 32
	conv0_2	3 × 3,32,S1	1/2H × 1/2W × 32
	conv0_3	3 × 3,32,S1	1/2H × 1/2W × 32
	conv1_x	$\left[\begin{array}{c} 3\times 3,32\\ 3\times 3,32 \end{array}\right]\times 3$	1/2H × 1/2W × 32
	conv2_x	$\left[\begin{array}{c} 3\times 3,64\\ 3\times 3,64 \end{array}\right]\times 16$	1/4H × 1/4W × 64
	conv3_x	$\left[\begin{array}{c} 3\times 3,128\\ 3\times 3,128 \end{array}\right]\times 3,dila=2$	1/4H × 1/4W × 128
	conv4_x	$\left[\begin{array}{c} 3\times 3,128\\ 3\times 3,128 \end{array}\right]\times 3,dila=4$	1/4H × 1/4W × 128
Attention module	SAM	Linear-attention	1/4H × 1/4W × 128
		Q:1 × 1,16,S1	
		K:1 × 1,16,S1	
		V:1 × 1,128,S1	
		E:Parameter(torch.Tensor(k,1/4H × 1/4W))	
		F:Parameter(torch.Tensor(k,1/4H × 1/4W))	
		1 × 1,64,S1	1/4H × 1/4W × 64
	CAM	Self-Attention	1/4H × 1/4W × 128
		1 × 1,64,S1	1/4H × 1/4W × 64
Construction of matching cost	Concat	[conv2_16,conv4_3,SAM,CAM]	1/4H × 1/4W × 320
	Fusion	3 × 3,128,S1	1/4H × 1/4W × 32
		1 × 1,32,S1	
	Concat	Left and shifted right	1/4D × 1/4H × 1/4W × 64
3D CNN aggregation	Preprocess
	conv1	[3 × 3 × 3,32,S1] × 2	1/4D × 1/4H × 1/4W × 32
	conv2	[3 × 3 × 3,32,S1] × 2	1/4D × 1/4H × 1/4W × 32
	output	ADD[conv1,conv2]	1/4D × 1/4H × 1/4W × 32
	Hourglass Module 1,2,3
	3Dstack x_1	3Dstack1a:3 × 3 × 3,64,S2	1/8D × 1/8H × 1/8W × 64
		3Dstack1b:3 × 3 × 3,64,S1	1/8D × 1/8H × 1/8W × 64
	3Dstack x_2	3Dstack2a:3 × 3 × 3,128,S2	1/16D × 1/16H × 1/16W × 128
		3Dstack2b:3 × 3 × 3,128,S1	1/16D × 1/16H × 1/16W × 128
	3Dstack x_3	deconv1^*^:3 × 3 × 3,64,S2	1/8D × 1/8H × 1/8W × 64
		shortcut1^*^:1 × 1 × 1,64,s1,3Dstack1b	1/8D × 1/8H × 1/8W × 64
		ADD[deconv1^*^,shortcut1^*^],ReLU	1/8D × 1/8H × 1/8W × 64
	3Dstack4 x_4	deconv2^*^:3 × 3 × 3,32,S2	1/4D × 1/4H × 1/4W × 32
		shortcut2^*^:1 × 1 × 1,32,s1,conv2	1/4D × 1/4H × 1/4W × 32
		ADD[deconv2^*^,shortcut2^*^],ReLU	1/4D × 1/4H × 1/4W × 32
Disparity prediction	conv1	3 × 3 × 3,32,S1	1/4D × 1/4H × 1/4W × 32
	conv2^**^	3 × 3 × 3,1,S1	1/4D × 1/4H × 1/4W × 1
	Upsample	Bilinear interpolation	D × H × W
	disparity	Soft Argmin	H × W

* Indicate that ReLU is not included.

** Indicate that ReLU and BN are not included, only convolution.

ResNet is adopted as the backbone network for feature extraction, and its construction is similar to PSMNet, with the half dilation settings and without the SPP pooling module. The first stage convolution layers conv0_1, conv0_2, and conv0_3 use three 3 × 3 convolution filters to cascaded to obtain shallow features, and the output feature map size is 1/2 of the original image. The second stage convolution layers conv1_x, conv2_x, conv3_x, and conv4_x basic residual blocks are used to learn to extract deep semantic features. Downsampling with stride 2 was used in conv0_1 and conv2_1, and dilated convolution is applied to enlarge the receptive field in conv3_x and conv4_x. The output feature map size is 1/4 of the original size after ResNet processing.

The AM attention module adaptively aggregates long-range contextual information to enhance the representation of features. It consists of two parts: SAM and CAM. SAM contains three parallel 1 × 1 2D convolutions for calculating Q, K, and V, and two linear projection layers E and F are used for dimensional-reduction processing of K and V. The size and channels of the calculated feature based on the Linear-Attention mechanism remain unchanged, then the number of channels is halved by a 1 × 1 2D convolution. CAM is calculated on the original feature map, and the size and channel of the feature after calculation based on the Self-Attention mechanism remain unchanged, then the number of channels is also halved by a 1 × 1 2D convolution.

The construction of a matching cost module cascades conv2_16, conv4_3, SAM, and CAM with low-level structural information, high-level semantic information, and global and local information to construct a 1/4H × 1/4W × 320 feature map. Two 2D convolution layers shared by weights were used to fuse the feature map and compress its channel to 32. A 4D matching Cost-volume of 1/4D × 1/4H × 1/4W × 64 was formed by connecting the left feature map of 2D and the right feature map under each disparity correspondence.

The 3D CNN Aggregation module aggregates semantic and structural feature information in the disparity dimensions and spatial dimensions to predict refined cost volumes, which contains two structures: basic structure and stacked hourglass structure. The basic structure is used to test the performance of each module. It consists of six 3D convolutional groups, each consisting of two 3D convolutional layers with stride 1, kernel size 3 × 3 × 3, BN and ReLU, and the output of each group is summed with the result of the next group, while the last group is not summed. The stacked hourglass structure is used to optimize the network. The pre-hourglass network in a stacked hourglass structure consists of four 3D convolutions with BN and ReLU. Three stacked 3D hourglass networks in stacked hourglass structures have the same architecture: 3Dstack x_1, 3Dstack ×_2, 3Dstack x_3, and 3Dstack x_4. Two downsamplings are performed during the 3D convolution encoding process, and two upsamplings are performed during the 3D deconvolution decoding process accordingly i.e., the 3D deconvolution with a convolution filter of 3 × 3 × 3 is used to recover the dimensions, while the number of channels is halved.

The disparity prediction module performs two 3D convolutions with a convolution kernel of 3 × 3 × 3 on each output unit of Output1, Output2, and Output3 to obtain a 4D volume of 1/4D × 1/4H × 1/4W × 1, applying trilinear interpolation to recover the same dimension H × W × D as the input image size, which is converted to a probability volume using a softmax function along the disparity dimension.

### Attention module

The SPP spatial pyramid pooling module in PSMNet expands the receptive field by using different scales of convolution to capture the global context information, but it does not further integrate and interact with the extracted features and lacks long-distance dependencies between information, so it cannot exploit the correlation between pixels to capture scene information. We designed an attention module AM, which models semantic relevance in the spatial and channel dimensions, respectively, captures long-range dependencies between global contexts, and adaptively integrates local and global information after feature extraction to obtain a better feature representation at the pixel level.

#### Spatial attention module

Spatial attention module encodes broader contextual information into local features from a global view of the entire feature map, adaptively aggregates information from the spatial environment, finds correlations between pixel features at different positions, and similar semantic features promote each other to improve intra-class cohesion and semantic consistency. Self-Attention in Transformer (Vaswani et al., 2017) is calculated as follows.


(1) $$\begin{array}{l} head_{i}=Attention\left(QW_{i}^{Q},KW_{i}^{K},VW_{i}^{V}\right)\\ \;=softmax\left[\frac{QW_{i}^{Q}{\left(KW_{i}^{K}\right)}^{T}}{\sqrt{d_{k}}}\right]\cdot \left(VW_{i}^{V}\right) \end{array}$$

where $P=softmax\left[\frac{QW_{i}^{Q}{\left(KW_{i}^{K}\right)}^{T}}{\sqrt{d_{k}}}\right]$, *P* ∈ ℝ^*n*×*n*^ is a context mapping matrix which represents the correlation of pixels at different positions. $Q,K\in {\mathbb{R}}^{n\times d_{k}},V\in {\mathbb{R}}^{n\times d_{v}}$ are the query matrix, key matrix and value matrix of input embedding, respectively, *Q* = *XW*^*Q*^, *K* = *XW*^*K*^, *V* = *XW*^*V*^, $X\in {\mathbb{R}}^{n\times d_{m}}$ is the input sequence, n is sequence length, *d*_*m*_ is the embedding dimension, *W*^*Q*^, $W^{K}\in {\mathbb{R}}^{d_{m}\times d_{k}}$, $W^{V}\in {\mathbb{R}}^{d_{m}\times d_{v}}$ are three learnable matrices and *d*_*m*_, *d*_*k*_, *d*_*v*_ are the hidden dimensions of the projection subspaces, for the rest of this article, we will not differentiate between *d*_*k*_ and *d*_*v*_ and just use d.

Self-Attention first calculates the dot product with *Q* ∈ ℝ^*n*×*d*^ and *K* ∈ ℝ^*n*×*d*^, after scaling and normalization the attention matrix *P* ∈ ℝ^*n*×*n*^ is obtained, and then fuses the values of V with the values of P. Since both Q and K are n × d dimensional matrices, the time complexity of multiplying the two is O(*n*^2^), and since the matrix P is an n × n dimensional matrix, the space complexity is also O(*n*^2^). Therefore, the cost of training and deploying the model when using Self-Attention on large size images is very high, even if the input image size is brought down by CNN, the time and space overhead it entails can significantly slow down the network. Therefore, A Linear-Attention is proposed, which can reduce the overall complexity of Self-Attention in time and space from O(*n*^2^) to O(n), without degrading performance and criteria, and at the same time with greater memory and time efficiency.


(2) $$\begin{array}{l} Linear-Attention\left(QW^{Q},KW^{K},VW^{V}\right)\\ \;=softmax\left[\frac{QW^{Q}{\left(EKW^{K}\right)}^{T}}{\sqrt{d}}\right]\cdot \left(FVW^{V}\right) \end{array}$$

**Figure 2:**
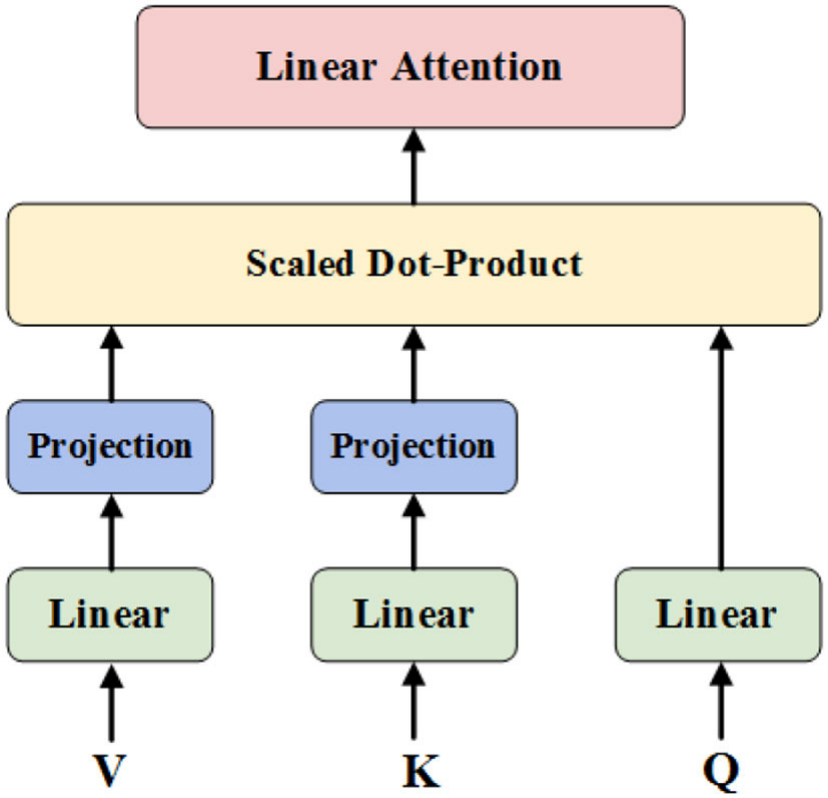
Linear-Attention architecture.

**Figure 3:**
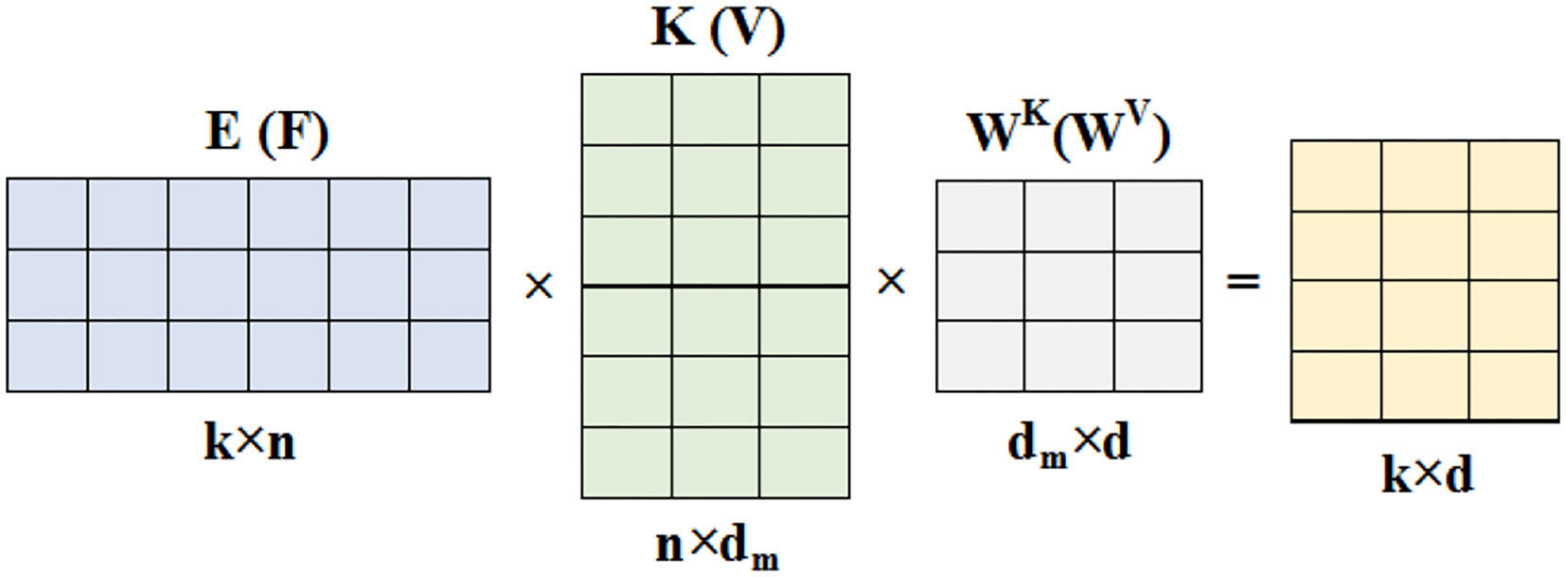
Linear mapping layers.

Where $\overset{-}{P}=softmax\left[\frac{QW^{Q}{\left(EKW^{K}\right)}^{T}}{\sqrt{d}}\right]$, *P* ∈ ℝ^*n*×*k*^. The contextual mapping *P* in Self-Attention is low-rank, and most of the information of matrix*P* is concentrated in the few largest singular values, P can be approximated by a low-rank matrix $\overset{-}{P}$, thus we can reduce the complexity of Self-Attention by changing its architecture. The main idea of the proposed Linear-Attention (Figure 2) is a low-rank approximation method, specifically adding two (k × n)-dimensional linear projection matrices E and F, respectively, when calculating Key and Value, which reduces the original Key and Value layer *KW*^*K*^ and *VW*^*V*^ from (n × d)-dimensional to (k × d)-dimensional. as in Figure 3. The (n × k)-dimensional contextual mapping matrix $\overset{-}{P}$ is then calculated by scaled dot-product, as in Equation (2).

According to the above operation, Linear-Attention has a value $\overset{-}{P}\cdot \left(FVW^{V}\right)$ and its time and space complexity is mainly O(nk) of $\overset{-}{P}$. If we choose a very small projected dimension k, such that *k* ≪ *n*, the overall complexity can be reduced to linear O(n). It can be proved that when *k* = *O*(*d*/ε^2^), $\overset{-}{P}\cdot \left(FVW^{V}\right)$ of Linear-Attention can be approximately equivalent to *P*·(*VW*^*V*^) of Self-Attention and the error is not greater than ε.

**Proof**. We first proof Self-Attention is low rank and then there exists matrices *E, F* ∈ ℝ^*n*×*k*^ that make Self-Attention linear complexity.

**Proof 1**. We define S and *D*_*S*_ as follows


(3) $$\begin{array}{l} S=\frac{{{QW}^{Q}\left({KW}^{K}\right)}^{T}}{\sqrt{d}} \end{array}$$

(4) $$\begin{array}{l} {\left(D_{S}\right)}_{ii}=\overset{N}{\underset{j=1}{\sum }}exp\left(S_{ji}\right) \end{array}$$

where *D*_*S*_ is an *n* × *n* diagonal matrix, the context mapping matrix P is defined as


(5) $$\begin{array}{l} P=softmax\left[\frac{{QW}^{Q}{\left(KW^{K}\right)}^{T}}{\sqrt{d}}\right]={exp\left(S\right)\cdot D}_{S}^{-1} \end{array}$$

define $\overset{-}{P}$ as follows with approximation error ε > 0


(6) $$\begin{array}{l} \overset{-}{P}={exp\left(S\right)\cdot D}_{S}^{-1}R^{T}R={PR}^{T}R \end{array}$$

Let *R* ∈ ℝ^*n*^ be a *k* × *n* matrix, 1 ≤ *k* ≤ *n*, with independent and identically distributed entries from *N*(0, 1/*k*), the rank of $\overset{-}{P}$ satisifies $rank\left(\overset{-}{P}\right)\leq rank\left(R\right)=k$.

According to the Johnson-Lindenstrauss lemma (JL, for short) (Arriaga and Vempala, 2006).


(7) $$\begin{array}{l} \text{P}r\left(\left(1-\varepsilon \right)\|u-v|{|}^{2}\leq \|u^{{\;}^{\prime }}-v^{{\;}^{\prime }}|{|}^{2}\leq \left(1+\varepsilon \right)\|u-v|{|}^{2}\right)\\ \geq 1-2e^{-\left(\varepsilon^{2}-\varepsilon^{3}\right)\frac{k}{4}}. \end{array}$$

For any row vector *x* ∈ ℝ^*n*^ of matrix P and any column vector *y* ∈ ℝ^*n*^ of matrix *VW*^*V*^, we can obtain


(8) $$\begin{array}{l} \text{P}r\left(\left\|{xR}^{T}{Ry}^{T}-{xy}^{T}\|\leq \varepsilon \|{xy}^{T}\right\|\right)>1-2e^{-\left(\varepsilon^{2}-\varepsilon^{3}\right)\frac{k}{4}}. \end{array}$$

furthermore, we have


(9) $$\begin{array}{l} \mathrm{Pr}\left(\left\|\overset{-}{P}y^{T}-Py^{T}\right\|\leq \varepsilon \left\|Py^{T}\right\|\right)=\mathrm{Pr}(\left\|PR^{T}Ry^{T}-Py^{T}\right\|\\ \;\leq \varepsilon \left\|Py^{T}\right\|)\\ \;=1-\mathrm{Pr}\left(\left\|PR^{T}Ry^{T}-Py^{T}\right\|>\varepsilon \left\|Py^{T}\right\|\right)\\ {\;}_{\geq }^{\left(I\right)}1-\sum_{x\epsilon P}\mathrm{Pr}\left(\left\|xR^{T}Ry^{T}-xy^{T}\right\|>\varepsilon \left\|xy^{T}\right\|\right)\\ \;\geq 1-\sum_{x\epsilon P}[1-\mathrm{Pr}\left(\left\|xR^{T}Ry^{T}-xy^{T}\right\|\leq \varepsilon \left\|xy^{T}\right\|\right)]\\ {\;}_{\geq }^{\left(II\right)}1-\sum_{x\epsilon P}\left[1-\left(1-2e^{-(\varepsilon^{2}-\varepsilon^{3})\frac{k}{4}}\right)\right]\\ \;>1-2ne^{-(\varepsilon^{2}-\varepsilon^{3})\frac{k}{4}} \end{array}$$

The above step (I) utilizes Boole's inequality


(10) $$\begin{array}{l} \mathrm{Pr}\left(\left\|{PR}^{T}Ry^{T}-Py^{T}\|>\varepsilon \|Py^{T}\right\|\right)\\ \leq \underset{x\in P}{\sum }Pr\left(\left\|xR^{T}{Ry}^{T}-xy^{T}\|>\varepsilon \|xy^{T}\right\|\right) \end{array}$$

Step (II) is based on the JL Lemma (8).

Therefore, we prove that for any *Q, K, V* ∈ ℝ^*n*×*d*^, *W*^*Q*^, *W*^*K*^, *W*^*V*^ ∈ ℝ^*d*×*d*^ and any column vector *y* ∈ ℝ^*n*^ of matrix *VW*^*V*^, when *k* = 5log(*n*)/(ε^2^ − *e*^3^), there exists a low-rank matrix $\overset{-}{P}\in {\mathbb{R}}^{n\times n}$, such that


(11) $$\begin{array}{l} \mathrm{Pr}\left(\left\|\overset{-}{P}y^{T}-Py^{T}\|<\varepsilon \|Py^{T}\right\|\right)\\ >1-o\left(1\right)and\;rank\left(\overset{-}{P}\right)=\Theta \left(log\left(n\right)\right) \end{array}$$

**Proof 2**.

Define *E* = δ*R* and *F* = *e*^−δ^*R*, where *R* ∈ ℝ^*k*×*n*^, 1 ≤ *k* ≤ *n*, with independent and identically distributed entries from *N*(0, 1/*k*), δ = 1/2^*n*^ is a constant, row vector *x* ∈ ℝ^*n*^ of matrix P and column vector *y* ∈ ℝ^*n*^ of matrix *VW*^*V*^, we have


(12) $$\begin{array}{l} {\left\|\exp\left(xE^{T}\right)Fy-exp(x)y^{T}\right\|}_{=}^{\left(I\right)}\left\|\exp\left(xE^{T}\right)Fy-exp(x)R^{T}Ry\right\|\\ \;+\left\|exp(x)R^{T}Ry-exp(x)y^{T}\right\|\\ {\;}_{=}^{\left(II\right)}\left\|\exp\left(x\delta R^{T}\right)e^{-\delta }Ry-exp(x)R^{T}Ry\right\|+\|exp(x)R^{T}Ry\\ \;-exp(x)y^{T}\|\\ {\;}_{\leq }^{\left(III\right)}(1+\varepsilon )\left\|\exp\left(x\delta R^{T}\right)e^{-\delta }y-exp(x)R^{T}y\right\|\\ \;+\left\|exp(x)R^{T}Ry-exp(x)y^{T}\right\|\\ {\;}_{\leq }^{\left(IV\right)}(1+\varepsilon )\left\|\exp\left(x\delta R^{T}\right)e^{-\delta }-exp(x)R^{T}\right\|\left\|y\right\|\\ \;+\left\|exp(x)R^{T}Ry-exp(x)y^{T}\right\|\\ {\;}_{\leq }^{\left(V\right)}o\left(\left\|exp(x)\right\|\left\|y\right\|\right)+\left\|exp(x)R^{T}Ry-exp(x)y^{T}\right\|\\ {\;}_{\leq }^{\left(VI\right)}o\left(\left\|exp(x)\right\|\left\|y\right\|\right)+\varepsilon \left\|exp(x)y^{T}\right\| \end{array}$$

In Equation (12), step(I) is Based on the triangle inequality, step (II) is the result of plugging in *E* = δ*R* and *F* = *e*^−δ^*R*, and step (III) is based on the JL Lemma (13)


(13) $$\begin{array}{l} Pr\left({\left\|u^{\prime }\right\|}^{2}\;\geq \;(1+\varepsilon ){\left\|u\right\|}^{2}\right)\leq e^{-(\varepsilon^{2}-e^{3})\frac{k}{4}} \end{array}$$

we have


(14) $$\begin{array}{l} Pr\left(\left\|Rx\right\|\leq (1+\varepsilon )\left\|x\right\|\right)>1-e^{-(\varepsilon^{2}-e^{3})\frac{k}{4}} \end{array}$$

Step (IV) utilizes the Cauchy inequality, the step (V) utilizes the fact that exponential function is Lipchitz continuous in a compact region. Then we can choose a small enough δ = θ(1/*n*), such that


(15) $$\begin{array}{l} \|\exp\left(\delta xR^{T}\right)-{exp\left(\delta x\right)R}^{T}\|=o\left(\left\|\exp\left(x\right)\right\|\right) \end{array}$$

Step (VI) is based on the JL Lemma (8), we have


(16) $$\begin{array}{l} \mathrm{Pr}\left(\left\|\exp(x)R^{T}Ry-\exp(x)y^{T}\right\|\leq \varepsilon \left\|\exp(x)y^{T}\right\|\right)\\ \;>1-2e^{-(\varepsilon^{2}-e^{3})\frac{k}{4}} \end{array}$$

Therefore, we prove that exist *E* = δ*R* and *F* = *e*^−δ^*R*, where*R* ∈ ℝ^*k*×*n*^, 1 ≤ *k* ≤ *n*, with independent and identically distributed entries from *N*(0, 1/*k*), δ = 1/2^*n*^ is a constant, for any row vector *x* ∈ ℝ^*n*^ of matrix P and any column vector *y* ∈ ℝ^*n*^ of matrix *VW*^*V*^, such that


(17) $$\begin{array}{l} \mathrm{Pr}\left(\left\|\exp\left(xE^{T}\right)Fy^{T}-\exp(x)y^{T}\right\|\leq \varepsilon \left\|\exp(x)y^{T}\right\|\right)\\ \;>1-2e^{-(\varepsilon^{2}-e^{3})\frac{k}{4}} \end{array}$$

Furthermore,


(18) $$\begin{array}{l} Pr(\|softmax\left(\frac{QW^{Q}{\cdot {(E\cdot KW}^{K})}^{T}}{\sqrt{d}}\right)\left(F\cdot VW^{V}\right)\\ \;-softmax\left(\frac{QW^{Q}{\cdot {(KW}^{K})}^{T}}{\sqrt{d}}\right)\left(VW^{V}\right)\| \end{array}$$

$$\begin{array}{l} \leq \varepsilon \left\|softmax\left(\frac{QW^{Q}{\cdot {(KW}^{K})}^{T}}{\sqrt{d}}\right)\left(VW^{V}\right)\right\|)>1-o(1) \end{array}$$

By setting *k* = 5log(*nd*)/(ε^2^ − *e*^3^), *where d* = *rank*(*S*)

Based on the above analysis, Linear-Attention features are calculated as follows. The output size of RestNet is 1/4H × 1/4W × 128, which is represented as the input feature of SAM as *X* ∈ ℝ^*H*×*W*×*C*^, feeding X into three 2D convolution layers of 1 × 1 to generate new feature maps *Q, K, V* ∈ ℝ^*H*×*W*×*C*^, respectively, *Q* = *XW*^*Q*^, *K* = *XW*^*K*^, *V* = *XW*^*V*^ and reshape them to *Q, K, V* ∈ ℝ^*n*×*d*^, where *n* = 1/4*H* × 1/4*W* and d=C. *E, F* ∈ ℝ^*k*×*n*^, according to the experiment in **Table 4**, we set the value of k to 512, and the performance of the model is the best at this value. In order to reduce the parameters of the network, let E and F share the parameters. We perform a matrix multiplication between *QW*^*Q*^ and the transpose of *EKW*^*K*^, after that a softmax layer is applied to calculate the spatial attention map $\overset{-}{P}\in {\mathbb{R}}^{n\times k}$.


(19) $$\begin{array}{l} {\overset{-}{P}}_{ji}=\frac{\exp\left[\frac{Q_{i}W_{i}^{Q}{\left(E_{j}K_{j}W_{j}^{K}\right)}^{T}}{\sqrt{d}}\right]}{\overset{n}{\underset{i=1}{\sum }}\exp\left[\frac{Q_{i}W_{i}^{Q}{\left(E_{j}K_{j}W_{j}^{K}\right)}^{T}}{\sqrt{d}}\right]} \end{array}$$

where ${\bar{P}}_{ji}\in {\mathbb{R}}^{n\times k}$ indicates the influence of position i on position j. A larger value of ${\overset{-}{P}}_{ji}$ indicates a greater correlation between the features of the two positions.

We perform a matrix multiplication between $\overset{-}{P}$ and *FVW*^*V*^ and reshape the result to *R*^*H*×*W*×*C*^, then multiply it by a scale parameter α and perform an element-wise sum operation with the original feature map of *X* ∈ ℝ^*H*×*W*×*C*^. Finally, we get the spatial attention feature map *Y* ∈ ℝ^*H*×*W*×*C*^.


(20) $$\begin{array}{l} Y_{j}=\alpha \overset{n}{\underset{i=1}{\sum }}\left({\overset{-}{P}}_{ji}F_{i}V_{i}W_{i}^{V}\right)+X_{j} \end{array}$$

Where α denotes the scale factor, which gradually learns a weight from 0 to get more weight. Equation (20) shows that the feature *Y*_*j*_ at each location is a weighted sum of the features at all locations and the original location*X*_*j*_. Thus, it has global contextual information and selectively aggregates contextual information according to the spatial attention map, and semantic features with high relevance promote each other and fuse similar features in the global space, which improves the compactness and semantic consistency within the features and plays an important role for feature representation and extraction in ill-posed regions.

#### Channel attention module

Each channel corresponds to a class-specific semantic feature map, which models the importance of individual feature channels and captures long-range semantic dependencies between channel features to improve the identification of channel features. Unlike the spatial attention mechanism, CAM does not involve O(*n*^2^) complexity and we use Self-Attention, the channel attention architecture is shown in Figure 1. Specifically, we reshape the input feature *X* ∈ ℝ^*H*×*W*×*C*^ to *Q*′, *K*′, *V*′ ∈ ℝ^*n*×*d*^ and, where *n* = 1/4*H* × 1/4*W* and d=C, then perform a matrix multiplication between the transpose of *Q*′ and *K*′. We apply a softmax layer to obtain the channel correlation matrix *P*′ ∈ ℝ^*d*×*d*^.


(21) $$\begin{array}{l} {P^{\prime }}_{ji}=softmax\left(\frac{{Q^{{\;}^{\prime }}}^{T}K^{{\;}^{\prime }}}{\sqrt{d}}\right)=\frac{exp\left(\frac{{Q^{{\;}^{\prime }}}_{i}^{T}K_{j}^{{\;}^{\prime }}}{\sqrt{d}}\right)}{\overset{C}{\underset{i=1}{\sum }}exp\left(\frac{{Q^{\prime }}_{i}^{T}K_{j}^{{\;}^{\prime }}}{\sqrt{d}}\right)} \end{array}$$

Where $P_{ji}^{\prime }$ denotes the influence of the ith channel on the jth channel, the more similar the features expressed by the two channels, the greater the response value between them. Matrix multiplication of *V*′ and $P_{ji}^{\prime }$ yields an ℝ^*n*×*d*^ feature map, which is reshaped to ℝ^*H*×*W*×*C*^ and then multiply the result by a scale parameter β and performs an element-wise sum operation with the original feature map of *X* ∈ ℝ^*H*×*W*×*C*^. Finally, we get the channel attention feature map *Z* ∈ ℝ^*H*×*W*×*C*^.


(22) $$\begin{array}{l} Z_{j}=\beta \overset{C}{\underset{i=1}{\sum }}\left(V_{i}^{{\;}^{\prime }}P_{ji}^{{\;}^{\prime }}\right)+X_{j} \end{array}$$

Where β denotes the scale factor, which is initialized to 0 and gradually learns to assign larger weights. It can be inferred from Equation (22), that the final feature Z of each channel is a weighted sum of the features of all channels and the original features, which ensures that the channel attention mechanism is able to capture the long-range semantic dependencies between channel features and obtain more contextual semantic information, and helps to improve the identification of features. The process of channel attention is similar to that of spatial attention, except that X is not processed before calculating the correlation matrix in the channel dimension, in order to maintain the original relationship between different channel maps. The feature of any two channels is directly multiplied by dimension transformation to obtain the correlation strength of any two channels. After softmax operation, the channel Attention matrix is obtained. Finally, Attention is fused by weighting, so that global correlation can be generated between all channels and stronger semantic response features can be obtained.

### 3D CNN aggregation

The 3D CNN Aggregation module aggregates semantic and structural feature information in the disparity dimensions and spatial dimensions to predict refined cost volumes. We use two 3D CNN structures for cost-volume regularization: the basic structure and the stacked hourglass structure. The basic structure is the same as PSMNet, and the stacked hourglass structure is optimized in this study. PSMNet uses a 3D stacked hourglass structure to aggregate multi-scale environmental information to achieve high matching accuracy, however, this 3D stacked hourglass structure has a lot of redundant information resulting in a large number of model parameters, high runtime cache usage and inefficient learning of the network. To solve this, the following modifications are made to reduce the number of parameters in the network and increase the inference speed of the network computation. The structure of the optimized stacked hourglass structure is shown in Figure 1 and Table 1.

First, Shortcut connections between different hourglass output modules have been removed: i.e., between output1 and output2 and between output2 and output3, so that the auxiliary output modules output1 and output2 can be removed during the inference process to save computational costs.

Second, A 1 × 1 × 1 3D convolution shortcut is used inside each hourglass module for direct connection, compared to a 3 × 3 × 3 3D convolution, the computational parameters of the network are reduced, and the number of calculations for its multiplication is reduced to 1/27 of the original, at which point it runs very fast and in negligible time, thus enabling the network to run faster without increasing the computational cost.

The optimized 3D CNN aggregation module consists of a pre-processing network and an hourglass network, with the pre-processing network used to extract low-level features and provide geometric constraints for disparity prediction. The hourglass network learns more semantic and structural information about the contextual environment and is able to refine the low-texture blur and occlusion parts, which are used to compute the final disparity map. The 3D stacked hourglass network uses an “encode-decoder” structure to solve the problem of over-computation caused by 3D CNNs, where the encoder uses a 3D convolution of step 2 to downsample and the decoder uses a step 2 deconvolution to recover size. In order to reduce the loss of spatial information caused by the “encoder-decoder” structure, we connect features of the same size corresponding to the encoder and decoder allows the lost detail information and information from the lower-level feature maps to be added during the process of deconvolution to recover the resolution of the feature maps.

### Disparity prediction

The three hourglasses correspond to three outputs and three losses. During the training phase, the total loss is obtained from the weighted sum of the three losses. During the testing phase, each hourglass output generates a disparity map, and the final disparity map is obtained from the last output. First, each value in the cost volume is transformed into a probability value p along the disparity dimension by using Softmax, then the disparity value k for each pixel is multiplied by the corresponding probability *p*_*k*_ and cumulative summed. Finally, the disparity estimation $\hat{d}$ is obtained by regression method using a differentiable Soft Argmin function (Kendall et al., 2017).


(23) $$\begin{array}{l} \hat{d}=\overset{D_{max}-1}{\underset{k=0}{\sum }}{k\cdot p}_{k} \end{array}$$

Where *D*_*max*_ indicates the maximum disparity. For each pixel, we have a D max -length vector which contains the probability p for all disparity levels. k and pk denote a possible disparity level and the corresponding probability.

Since the smoothed L1 loss function is more robust and less sensitive to abnormal values than the L2 loss function, the L1 loss function is widely used in object detection for bounding box regression, and the disparity calculation can also be considered as a regression problem, so we use the L1 loss function (Goodfellow et al., 2016). The total loss was calculated as follows.


(24) $$\begin{array}{l} L=\overset{3}{\underset{i=1}{\sum }}\lambda_{i}\cdot Smooth_{L_{1}}\left({\hat{d}}_{i}-d_{i}\right) \end{array}$$

in which


(25) $$\begin{array}{l} Smooth_{L_{1}}(x)=\{\begin{array}{c} 0.5x^{2},\;if|x|<1\\ |x|-0.5,\;otherwise \end{array} \end{array}$$

Where λ_*i*_ denotes the coefficient of the *i*^*th*^ disparity prediction, *d*_*i*_ denotes the *i*^*th*^ ground truth disparity map and ${\hat{d}}_{i}$ denotes the *i*^*th*^ prediction disparity map.

## Data and performance evaluation metrics

**Figure 4:**
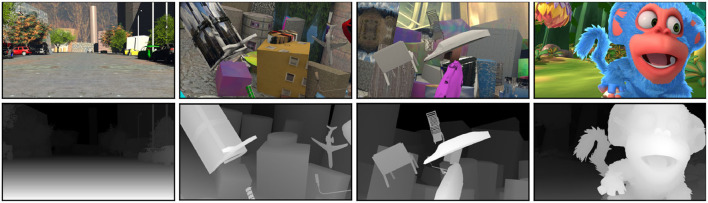
Visualization of scene flow dataset.

The Scene Flow dataset (Mayer et al., 2016) is a large-scale public dataset of synthetic non-real scenes applied to binocular stereo matching, which is created through computer graphics rendering techniques and provides dense ground-truth disparity maps for all image pairs. As shown in Figure 4. It contains three sub-datasets: Flyingthings 3D, Monkaa and Driving, with a total of 39,049 pairs, of which 34,801 training image pairs and 4,248 test image pairs. In this study, 90% of the training image pairs are used as the training set, and 10% are used as the validation set. The details are listed in Table 2.

**Table 2:** Details of scene flow dataset.

**Subset**	**Training set**	**Test set**	**Total**	**Resolution**	**Sparse/Dense**	**Synthetic/Real**
Flying things 3D	21,818	4,248	26,066	960^*^540	Dense	Synthetic
Driving	4,392	—	4,392	960^*^540	Dense	Synthetic
Monkaa	8,591	—	8,591	960^*^540	Dense	Synthetic

Figure 4 is a display of four samples. The images in the first row are the left images of the Scene Flow dataset, the images in the second row are the right images of the Scene Flow dataset, and the images in the third row are the disparity images corresponding to the left images.

**Figure 5:**
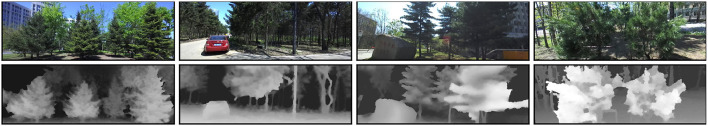
Visualization of forest dataset.

Forest is a dataset of real Forest scenes that we created ourselves, which were collected from the forestry field of Northeast Forestry University. As shown in Figure 5. The acquisition device is a ZED2 binocular depth camera with a pixel resolution of 1,280^*^720, which can acquire binocular image pairs and their corresponding disparity maps simultaneously. The original image pairs and their corresponding disparity maps are cropped into images with a resolution of 1240*426 to form the forest dataset, of which 80% are used as the training set, 10% as the validation set, and 10% as the test set. Forest dataset contains a total of 400 pairs of binocular image pairs and their corresponding dense disparity maps of five types of forest vegetation, including Larix gmelinii, Pinus sylvestris var. mongolica, Pinus tabulaeformis var. mukdensis, Fraxinus mandschurica Rupr, and Betula platyphylla Suk. The details are listed in Table 3.

**Table 3:** Layers and parameter settings of the proposed LANet.

**Variety**	**Training set**	**Validation set**	**Test set**	**Total**	**Resolution**	**Sparse/Dense**	**Synthetic/Real**
Larix gmelinii	72	9	9	90	1,240*426	Dense	Real
Pinus sylvestris var. mongolica	72	9	9	90	1,240*426	Dense	Real
Pinus tabulaeformis var. mukdensis	64	8	8	80	1,240*426	Dense	Real
Fraxinus mandschurica Rupr	56	7	7	70	1,240*426	Dense	Real
Betula platyphylla Suk	56	7	7	70	1,240*426	Dense	Real

The recognized performance evaluation metrics in the binocular stereo matching task are as follows.

(1) End-point-error

End-point-error (EPE) represents the average Euclidean distance between the predicted disparity and the true disparity of a pixel.


(26) $$\begin{array}{l} EPE=\frac{1}{N}\underset{i\epsilon N}{\sum }\sqrt{{\left(d_{i}-{\hat{d}}_{i}\right)}^{2}} \end{array}$$

where N denotes the total number of pixel points, *d*_*i*_ denotes the true disparity of the *i*^*th*^ pixel, and ${\hat{d}}_{i}$ denotes the predicted disparity of the *i*^*th*^ pixel.

(2) T-pixel-error

T-pixel-error indicates that the absolute value of the difference between the predicted disparity and the true disparity exceeds the number of t pixel points as a percentage of the number of pixels in the whole image, the higher the percentage the more false matching points and the lower the matching accuracy.

(3) D1-all

D1-all is the percentage of pixels with errors of more than three pixels or 5% of disparity error from all test images.

(4) Parameter

The parameter is the total number of parameters for model training, in millions.

(5) Runtime

Runtime is the average running time to generate a disparity map, in seconds.

## Experimental results and discussion

### Experimental detail setting

The current mainstream training method for binocular stereo matching algorithms is to pre-train on Scene Flow and then fine-tune on the target dataset, which can achieve better results on the target dataset. In this study, the pre-training dataset uses the clean pass in Scene Flow and its corresponding disparity map of the left image, and the target dataset uses Forest and its corresponding disparity map of the left image, the detailed setup of the experiment is shown below.

The network was based on Python 3.9.7, the PyTorch 1.11.0 framework, and the optimizer used Adam (Diederik and Ba, 2015) with β_1_= 0.9 and β_2_= 0.999. The network model was trained on an Nvidia TITAN Xp GPU 3090 with batch size set to 8 and the coefficients for the three outputs set to λ1= 0.5, λ2= 0.7, and λ3 = 1.0, respectively.

The model was trained from scratch on the Scene Flow dataset for 16 epochs, with an initial learning rate of 0.001. After the 10th epoch, the learning rate decayed by half every 2 epochs, ending at 0.000125, and the training process took about 19 h. During training, images were randomly cropped to size H = 256 and W = 512. The maximum disparity (D) was set to 192. The model trained on Scene Flow was used directly in the ablation experiments. A full image of size 960 × 540 was directly fed to the network for disparity prediction, and during the network evaluation, we removed “invalid” images with less than 10% of valid pixels (0 ≤ *d* < *D*_*max*_) from the test set, only “valid” images were tested. The pre-trained model on Scene Flow was fine-tuned on Forest for 800 epochs, with the initial learning rate of 0.001 and the learning rate decaying by half every 200 epochs, ending at 0.000125. The fine-tuning took about 12 h to obtain the final model, which was used to evaluate the final accuracy and effectiveness of the model.

### Results

#### Results of experiments on scene flow

(1) Ablation experiments on Scene Flow

In this section, ablation experiments are conducted on the Scene Flow dataset to verify the performance of each key module and key parameters in LANet. In Table 2, Res is the ResNet module, CAM is the channel attention module, SA is a spatial attention module using the Self-Attention mechanism, SAM is a spatial attention module using the Linear-Attention mechanism, kx is the dimension of E in the model and E and F share parameters, Basic is the basic structure and Hourglass is the stacked hourglass network. The performance of the CAM and SAM modules is evaluated by using the basic structure since Basic does not learn additional contextual information through an “encoder-decoder” process. The performance of each module was evaluated by one-pixel-error (>1px), two-pixel-error (>2px), and three-pixel-error(>3px) errors and EPE and Runtime, respectively, and the experimental results are shown in Table 4.

**Table 4:** Ablation experiments on scene flow.

**Model**	**>1px(%)**	**>2px(%)**	**>3px(%)**	**EPE(px)**	**Runtimes(s)**
Res_Base	12.78	8.11	6.41	1.65	0.12
Res_CAM_Base	11.12	7.02	5.36	1.21	0.14
Res_SA_Base	10.24	6.48	4.91	1.03	0.24
Res_SAM_k128_Base	10.47	6.65	5.04	1.10	0.16
fRes_SAM_k256_Base	10.38	6.58	4.98	1.07	0.17
Res_SAM_k512_Base	10.29	6.52	4.93	1.05	0.18
Res_CAM_SAM_k512_Base	9.26	5.56	3.95	0.95	0.19
Res_CAM_SAM_k512_Hourglass	7.22	3.71	2.31	0.82	0.25

It can be seen from Table 4 that CAM and SAM were added to significantly reduce the error rate compared to Res_Base, the EPE decreased from 1.65 to 1.21 and 1.10 of k128, respectively, and three-pixel-error (>3px) decreased from 6.41 to 5.36 and 5.04 of k128, respectively. It is demonstrated that the attention modules CAM and SAM help to reduce the false match rate. In order to examine the performance of Linear-Attention, the Res_SA_Base module is added for comparison, and k is set to different values. When k=128, the EPE of Res_SAM_k128_Base is 1.10 higher than 1.03 of Res_SA_Base, but the inference time of Res_SAM_k128_Base is 0.16s, which is much lower than 0.24s of Res_SA_Base. When the value of k increases, the EPE of Res_SAM_kx_Base gradually approaches that of Res_SA_Base. When k=512, the EPE of the two is almost the same, while the inference time of Res_SAM_kx_Base does not change much and is significantly faster than that of Res_SA_Base. This verifies that when the error rates of the two are close, the speed of linear-attention is significantly faster than that of self-attention. Res_CAM_SAM_k512_Base has obvious advantages over Res_CAM_SAM_k512_Hourglass, which reduces the three-pixel-error of the overall network from 3.95 to 2.31 and EPE from 0.95 to 0.82.

(2) Comparison experiments with other methods on Scene Flow

In order to examine the performance of the model, LANet is compared with the state-of-the-art methods such as Edgestereo, GC-Net, and PSMNet on the Sceneflow test set from the three performance evaluation metrics of three-pixel-error, EPE and Parameter.

As listed in Table 5, the three-pixel-error of each model is basically in proportion to its EPE, but there is no direct relationship with the number of parameters. The number of parameters in GCNet is 3.5M, which is small, but its error rate is higher. Due to a large number of convolution layers in the CRL, the number of parameters is up to 78.77M, and the model is bloated and inefficient. The PSMNet shows a good performance in all metrics, while LANet shows better.

**Table 5:** Comparison experiments on scene flow.

**Method**	**>3px**	**EPE**	**Parameter**
	**(%)**	**(px)**	**(**10^6^)****
MC-CNN (Zbontar and LeCun, 2015)	13.70	3.79	—
GCNet (Kendall et al., 2017)	9.34	2.51	3.50
iResNet (Liang et al., 2017)	4.64	2.46	43.11
DispNet (Mayer et al., 2016)	9.27	1.68	42.00
CRL (Pang et al., 2017)	6.20	1.32	78.77
SegStrreo (Yang et al., 2018)	4.74	1.77	—
EdgeStereo (Song et al., 2020)	4.35	1.45	—
PSMNet (Chang and Chen, 2018)	2.43	1.09	5.20
LANet(Ours)	2.31	0.82	4.50

(3) Visualization of Scene Flow

**Figure 6:**
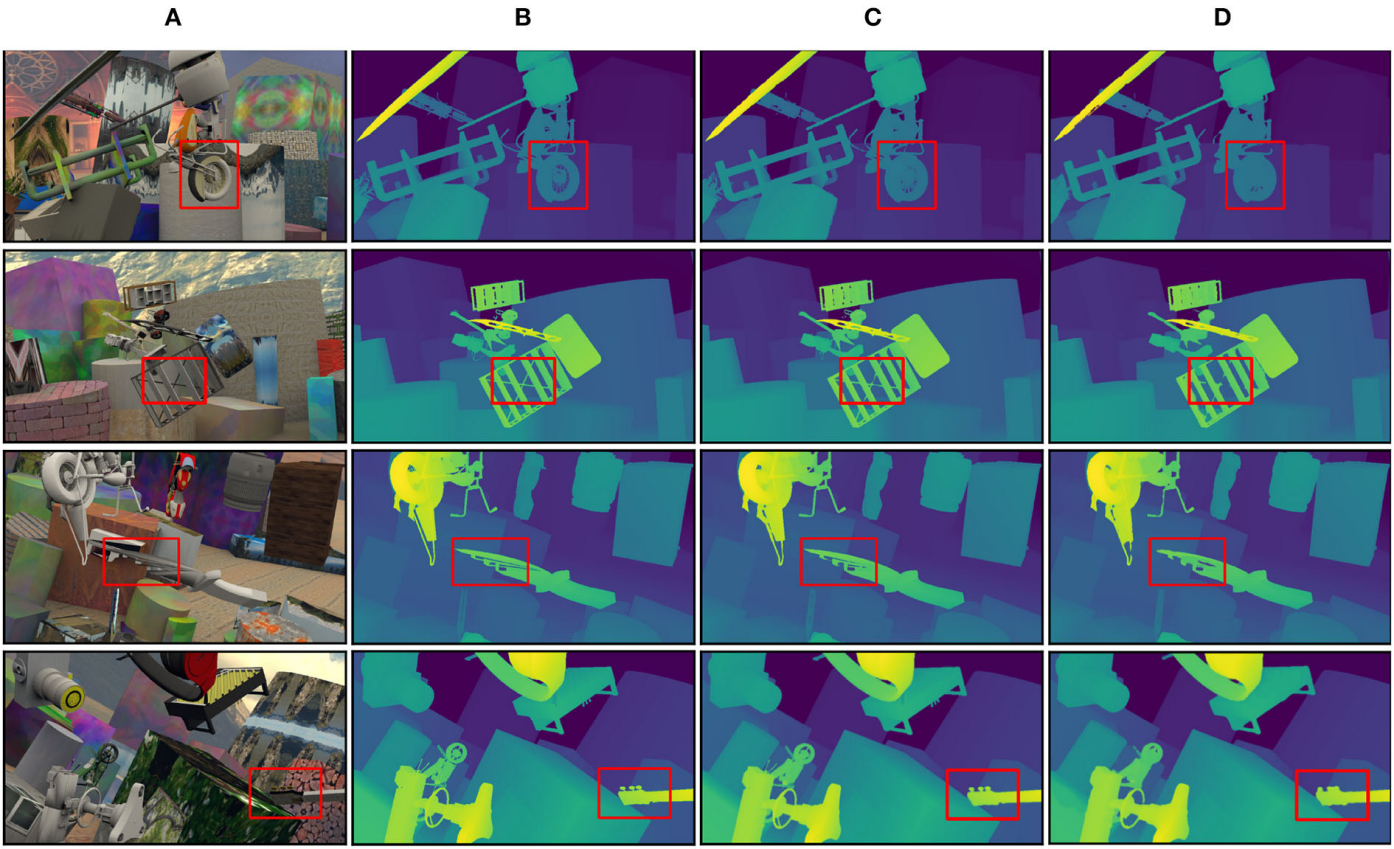
Visualization on scene flow. **(A)** Left images, **(B)** ground truth, **(C)** LANet, and **(D)** PSMNet.

Figure 6 illustrates some examples of the disparity maps estimated by the proposed LANet and PSMNet on Scene Flow. Where the first column is the left images of Scene Flow, the second column is the ground truth, the third column is the disparity maps estimated by LANet, and the fourth column is the disparity maps estimated by PSMNet.

The red rectangle in Figure 6 is all fine structure areas. It can be observed that the disparity effect is more obvious in the complex and precise areas of the “wheels” in the first group and the repetitive texture areas of the “tool” in the fourth group. LANet performs relatively well in these areas, not only retaining the complex and fine features of the “wheel” but also obtaining more reliable disparity maps in the “tool” repetitive texture area and the “shelf” occluded area. For objects with large and regular, more accurate matching can be achieved.

#### Results of experiments on Forest

(1) Setting of loss function weights

Properly setting the loss weights for each output module enables effective error return from the front to back of the entire network, which helps to improve network performance effectively. The stacked hourglass of the 3D CNN has three training outputs out1, out2, and out3 corresponding to three loss of weights λ_1_, λ_2_, and λ_3_, which are assigned a value between 0 and 1. In order to find the best loss weight, experiments with different weight combinations are designed and applied to the verification set of Scene Flow and Forest, as shown in Table 6.

**Table 6:** Setting of weighting factors.

**Loss weight**			**Forest**	**Scene flow**
**λ_1_**	**λ_2_**	**λ_3_**	**val EPE(px)**	**val EPE(px)**
0.0	0.1	1.0	1.25	1.53
0.1	0.3	1.0	1.06	1.34
0.3	0.5	1.0	0.91	1.01
0.5	0.7	1.0	0.68	0.82
0.7	0.9	1.0	0.85	0.93
1.0	1.0	1.0	0.94	1.04

The loss weights nearer to the end of the network are more important to the training of the network, so we set them relatively large, and the outputs of other modules play a supporting role in the training of the network. The best performance is obtained when the weights of λ_1_, λ_2_, and λ_3_ are 0.5, 0.7, and 1.0, respectively when the EPE is 0.82 on Scene Flow and 0.68 on Forest. For the basic structure, we treat these three loss weights equally and set them to 1.

(2) Comparison of model performance on Forest

In this group of experiments, three performance evaluation metrics, D1-all, EPE and Runtime, were used to examine the performance of each method on the Forest dataset. The results are shown in Table 7.

**Table 7:** Comparison experiments on Forest.

**Method**	**D1-all(%)**	**EPE(px)**	**Runtime(s)**
MC-CNN (Zbontar and LeCun, 2015)	4.08	3.96	67.09
GCNet (Kendall et al., 2017)	3.65	2.79	1.01
iResNet (Liang et al., 2017)	3.58	2.73	0.20
DispNet (Mayer et al., 2016)	3.08	1.96	0.14
CRL (Pang et al., 2017)	2.75	1.54	0.55
SegStrreo (Yang et al., 2018)	3.12	2.01	0.68
EdgeStereo (Song et al., 2020)	2.81	1.68	0.40
PSMNet (Chang and Chen, 2018)	2.61	1.25	0.48
LANet (Ours)	2.15	0.68	0.35

After fine-tuned on Forest, LANet showed better performance than that on the SceneFlow dataset, with an EPE reduction from 0.82 to 0.68. LANet was tested on a 3090 GPU and Forest dense test set, with an image resolution of 1240*426. The runtime was closely related to the performance of the experimental device and the size and density of the image, under our experimental conditions, the D1-all, EPE and runtime of PSMNet are 2.61, 1.25 and 0.48, respectively, while those of LANet are 2.15, 0.68 and 0.35, respectively, which are better than the baseline model PSMNet. In terms of Runtime, iResNet is 0.2 and DispNet is 0.14, which is better than our 0.35, but their accuracy is very low, which D1-all is 3.58 and 3.08, respectively, while ours is 2.15.

(3) Model visualization of Forest

**Figure 7:**
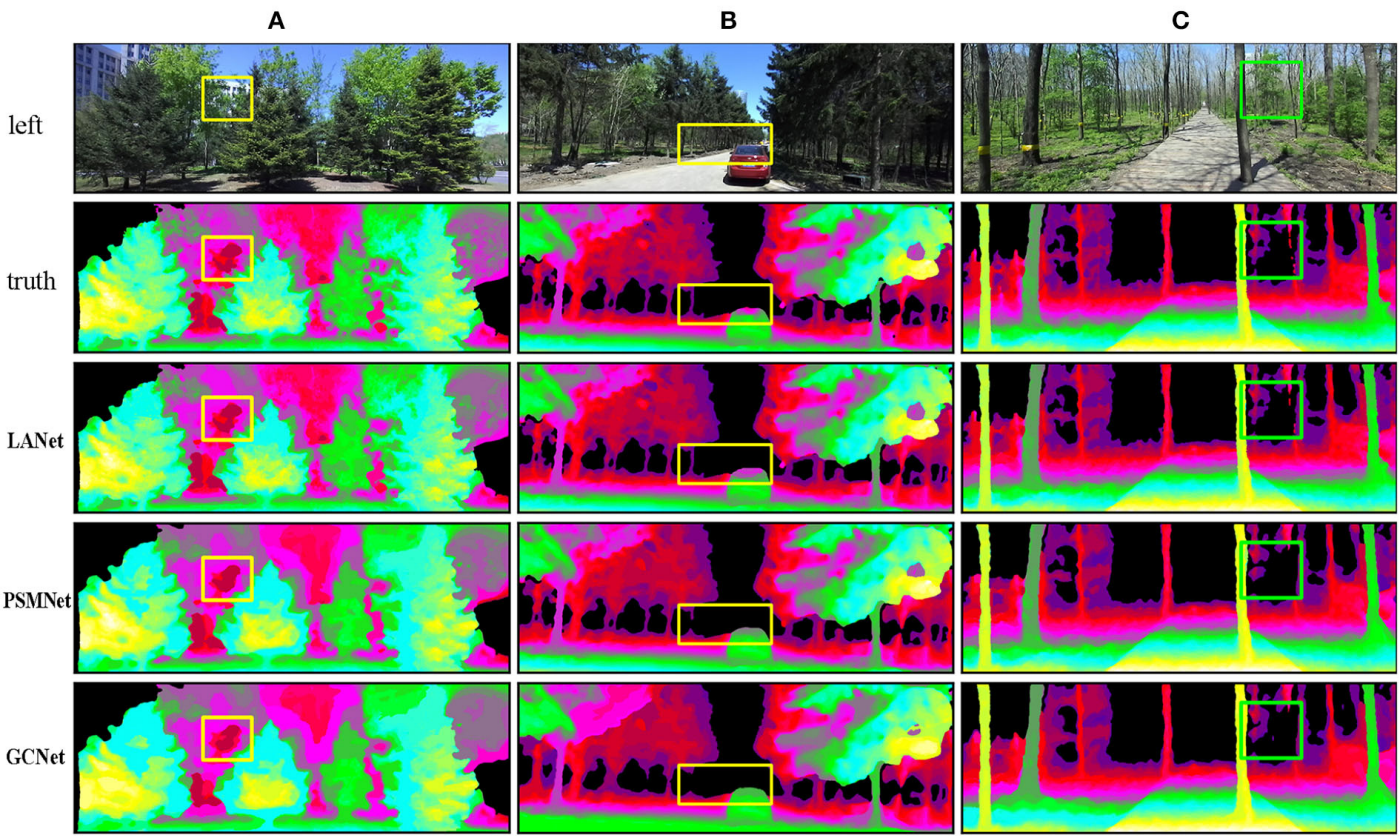
Visualization on forest.

Figure 7 shows the visualization of disparity maps generated by LANet, PSMNet, and GCNet on Forest, with color representing the different disparity values and black indicating points where the disparity values are very small and can be ignored at longer distances.

The yellow and green rectangular are areas of poor model matching, usually found in locations containing delicate and intricate structures such as branches, tree trunks, leaf edges, and weakly textured areas such as the rear glass of a car and obscured locations. In column A, the pink part of the yellow rectangular area shows the trees in the distance and the dark red part show the sky, with significant differences in predictions between models at the borders of the trees and the sky. PSMNet and GCNet can keep the main outline of the edge, but the matching of the delicate and intricate structure is not accurate enough, while LANet can keep the delicate and intricate features of the edge better and the prediction is closer to the true value. In column B, the yellow rectangular area is the distant tree trunk and the rear glass of the car. For the prediction of the red trunk, PSMNet and GCNet appear pixel missing, while the prediction of LANet is more accurate. For the pink rear glass of the car, LANet has a slight depth color deviation, while PSMNet has more depth color deviation and GCNet has more depth color error. In column C, the green rectangular area is the trunks and leaves in the distance. With LANet showing pixel discontinuities and a little missing for the red trunks, PSMNet and GCNet show larger pixel missing or even trunks missing. For the purple leaf part, LANet can better retain edge features, while PSMNet lacks some small edge features, and GCNet has too many edge predictions, resulting in a mismatch.

## Discussion

The proposed network was evaluated on two stereo-matched datasets, Scene Flow and Forest. Ablation experiments are conducted to verify the performance of each key module and key parameters in LANet. The results in Table 4 demonstrated that CAM and SAM significantly help to reduce the false match rate, and SAM performs better than CAM. The performance of the hourglass is significantly better than that of the Basic, indicating that the stacked hourglass module can better aggregate the feature information of disparity dimension and spatial dimension than the basic module, thus further improving the matching accuracy. The comparison between Res_SA_Base and Res_SAM_kx_Base verifies that when the error rates of the two are close, the speed of linear-attention is significantly faster than that of self-attention. Through the three sets of experiments of Res_SAM_kx_Base, it is found that when the k value becomes larger, their EPE gradually approaches that of Res_SA_Base, while the inference time does not change much, and is significantly faster than that of Res_SA_Base. When k = 512, the effect is the best, therefore, we choose k = 512 as the subsequent experimental parameters.

In order to investigate the overall performance of LANet, we also conducted experimental comparisons with other state-of-the-art models. As shown in Table 5, benefiting from the effective design of the attention mechanism and the refinement of the matching cost aggregation stage, with a three-pixel-error of 2.31, EPE of 0.82 and parameter number of 4.5M on Scene Flow, LANet achieves higher accuracy than PSMNet in the case of fewer parameters. The results in Table 7 show that the EPE of LANet is reduced from 0.82 to 0.68 after fine-tuning on Forest, and the accuracy is further improved, making it outperform the comparison model in terms of accuracy. The D1-all and running time of LANet are 2.15 and 0.35, respectively. Although its speed is not the fastest, combined with the above metrics, the comprehensive performance of LANet is very competitive.

Four testing examples on Scene Flow are illustrated in Figure 6 to demonstrate that LANet obtains relatively accurate disparity maps for delicate and intricate objects and overlapping occlusion objects. LANet can not only retain the delicate and intricate features of the “wheel” but also obtain more reliable disparity maps in the “tool” repetitive texture area and the “shelf” occluded area. Three testing examples on Forest shown in Figure 7 illustrate that LANet achieves more robust results in ill-posed regions. By comparison with PSMNet and GCNet, LANet performs relatively well, not only retaining the salient information of the object (e.g., branches, leaf edges, edge regions of tree trunks) but also still being able to extract valid features for more accurate matching in large weakly textured regions (e.g., glass, sky, roads) and obscured regions. The comparison model, however, has insufficient ability to identify valid features in these ill-posed regions due to the lack of interaction between global and local information, which can produce some false matches and affect the matching accuracy of the model. In addition, Although the comparison methods achieved high performance metrics on the target test set, they generally use fewer dataset samples during fine-tuning (e.g., KITTI only used 200 pairs), and many methods suffered from severe overfitting and poor generalization performance, resulting in an unsatisfactory performance on the Forest dataset.

Figures 6, 7 are the visualization results of the experimental comparison of various methods. The clarity of Figure 6 is high, and many details can be seen clearly, while Figure 7 is not as clear as Figure 6. The reasons for that are analyzed as follows. Figure 6 is an artificially synthesized close-up dataset, and the author has done some optimization processing on the dataset for binocular stereo matching to make the clarity of the figure higher, so we can see many details clearly. In contrast, Figure 7 is our own dataset of real inter-forest scenes. Because of the long distance and complex object structure of the field scenes, coupled with the fact that the authors did not optimize the dataset, Figure 7 does not look as clear as Figure 6, but this does not affect the accuracy of the model.

The results further show that the proposed attention mechanism can effectively identify salient features and fine structure features of different objects by capturing global long-range dependencies and aggregating rich global and local information, so as to extract more comprehensive and effective features to reduce matching errors and improve the disparity prediction accuracy. Robust results can still be obtained in some delicate and intricate regions, overlapping occlusion regions and other ill-posed regions, generating dense and reliable disparity maps for Forest scenes.

## Conclusion

This research makes full use of the global and local information of the forest scene environment to find consistent correlations in the ill-posed areas and proposes an end-to-end stereo matching network LANet, which uses the attention mechanism to better compensate for the shortage of convolutional receptive field and the lack of long-distance dependence of context information in PSMNet. The proposed Linear-Attention can significantly enhance the representation of contextual semantic features while reducing the computational complexity of Self-Attention from O(*n*^2^) to O(n), which will help improve the accuracy and speed of the network. The optimized 3D stacked hourglass aggregation network reduces the inference time and further improves the speed of the network. LANet achieves better accuracy than some state-of-the-art methods on SceneFlow and Forest, and obtains more robust results in delicate and intricate regions, overlapping occlusion regions and other ill-posed regions, generating dense and reliable disparity maps of the inter-forest scene, which will provide key data for 3D reconstruction of forest scenes. While the generalization performance of LANet on other datasets is to be further tested. In addition, in order to make the model have stronger learning ability and better robustness so that it can better adapt to the complex outdoor forest operation scenes, the number and variety of samples in the Forest dataset need to be expanded, and the quality of original disparity maps requires to be further improved, which will be an important study in the future research.

## Data availability statement

The raw data supporting the conclusions of this article will be made available by the authors, without undue reservation.

## Author contributions

LL proposed a constructive approach to stereo matching network, contributed to the construction of LANet, and program implementation and draft writing. YLiu contributed to the conceptualization, methodology, draft writing and reviewing, and provided experimental conditions, including the artificial intelligence laboratory and experimental equipment. YLv contributed to the draft writing, design and analysis of experiments, article editing and revision. JX contributed to conceptualization, methodology, and project support from the National Natural Science Foundation of China. All the authors contributed to the article and approved the submitted version.

## Funding

This research was funded by Fundamental Research Funds for the Central Universities (No. 2572021AW08), Cultivating an Excellent Doctoral Dissertation in Forestry Engineering (No. LYGCYB202008), and the National Natural Science Foundation Grant of China (No. 61975028).

## Conflict of interest

The authors declare that the research was conducted in the absence of any commercial or financial relationships that could be construed as a potential conflict of interest.

## Publisher's note

All claims expressed in this article are solely those of the authors and do not necessarily represent those of their affiliated organizations, or those of the publisher, the editors and the reviewers. Any product that may be evaluated in this article, or claim that may be made by its manufacturer, is not guaranteed or endorsed by the publisher.
